# Is Order the Defining Feature of Magnitude Representation? An ERP Study on Learning Numerical Magnitude and Spatial Order of Artificial Symbols

**DOI:** 10.1371/journal.pone.0049565

**Published:** 2012-11-19

**Authors:** Hui Zhao, Chuansheng Chen, Hongchuan Zhang, Xinlin Zhou, Leilei Mei, Chunhui Chen, Lan Chen, Zhongyu Cao, Qi Dong

**Affiliations:** 1 State Key Laboratory of Cognitive Neuroscience and Learning, Beijing Normal University, Beijing, P.R. China; 2 Department of Psychology and Social Behavior, University of California Irvine, Irvine, California, United States of America; 3 School of Social Development, Central University of Finance and Economics, Beijing, P.R. China; University of Groningen, The Netherlands

## Abstract

Using an artificial-number learning paradigm and the ERP technique, the present study investigated neural mechanisms involved in the learning of magnitude and spatial order. 54 college students were divided into 2 groups matched in age, gender, and school major. One group was asked to learn the associations between magnitude (dot patterns) and the meaningless Gibson symbols, and the other group learned the associations between spatial order (horizontal positions on the screen) and the same set of symbols. Results revealed differentiated neural mechanisms underlying the learning processes of symbolic magnitude and spatial order. Compared to magnitude learning, spatial-order learning showed a later and reversed distance effect. Furthermore, an analysis of the order-priming effect showed that order was not inherent to the learning of magnitude. Results of this study showed a dissociation between magnitude and order, which supports the numerosity code hypothesis of mental representations of magnitude.

## Introduction

Number, magnitude, and order are three closely related but distinct concepts. For example, a person's telephone number implies neither magnitude nor order. Similarly, numerical magnitude can be comprehended without number words as shown by animal research (see a review by Brannon [1]) and human infant research [e.g., [2], [3], [4], [5], [6]]. Finally, items can be ordered spatially or temporally or according to many other attributes. There is a longstanding debate, however, on whether the representation or processing of numerical magnitude is independent of order (especially spatial order). The two rival hypotheses are the mental number line hypothesis and the numerosity code hypothesis. The mental number line hypothesis proposes that numbers are represented as an ordered sequence of input nodes on an oriented analogical number line [e.g., [7], [8], [9]]. According to this hypothesis, the spatial order of numbers is inherent to, and inseparable from, the magnitude representation. In contrast, the numerosity code hypothesis [9] states that numbers' magnitudes are represented as the quantity of units (i.e., 5 represents five units), which does not require spatial order. In order to understand mental representations of numbers, we need to directly test these two hypotheses and to determine whether magnitude representation depends on order.

Several lines of previous research have shown that order and magnitude share similar representations. For example, behavioral research has shown that both number magnitude comparison tasks and tasks involving ordinal materials such as letters [10], [11] and months [10] show the distance effect, namely, subjects react faster and more accurately when discriminating stimuli that are closer to each other in either magnitude or serial position. A close relation between magnitude and order has also been supported by the classic SNARC (Spatial-Numerical Association of Response Code) effect [12], namely, an reaction time advantage in response to small numbers with the left hand and large numbers with the right hand. Finally, brain imaging data have shown that both numerical magnitude processing and order (spatial as well as temporal and serial order) processing activate the parietal regions [13], [14], [15], [16], [17], [18].

On the other hand, several studies have found evidence for a dissociation between magnitude and order processing. In a neuropsychological study of the patient CO [19] who suffered from acalculia due to damages to bilateral parietal regions, researchers found that CO could quickly and correctly perform a magnitude judgment task (i.e., Which of two numbers is smaller (or larger)?), but could not answer whether a number came before or after “5”. He also had difficulties with other tasks involving ordered series, such as letters, days and months. In contrast, the patient SE [20] who suffered a bilateral frontal infarct could count and answer the question of “which number comes next”, but showed difficulties in quickly identifying numerosity of a dot pattern and in performing number-comparison tasks. He had to resort to using his fingers or a counting strategy for the latter tasks. Furthermore, he showed a reversed distance effect during the number-comparison task. Beyond patient data, some electroencephalographic studies also revealed different activation patterns of magnitude processing and order processing. Szűcs and Csépe [21] found different patterns of the distance effect in the right parietal N2P for magnitude (numerosity judgment) and order processing (letter judgment). Specifically, far-distance pairs elicited more negative potentials than close-distance pairs in numerosity judgment, but the reverse (greater negative potentials for the close-distance pairs) was true for letters. Turconi [22] asked subjects to perform a magnitude task (comparing numbers with “15” in magnitude), an order-of-numbers task (determining numbers as before or after “15”), and an order-of-letters task (determining letters as before or after M). The distance effect appeared earlier on the P2p component and was left lateralized for magnitude processing, but was delayed and bilateral for order processing.

As shown by the above review of the literature, research evidence on the association between magnitude and order is inconsistent. One possible reason is that previous research used natural numbers, which may automatically activate multiple attributes such as their cardinality and ordinality, and comparison materials such as letters or months, which cannot be strictly matched with numbers. One way to avoid this potential weakness is to use artificial symbols as the material.

**Figure 1:**
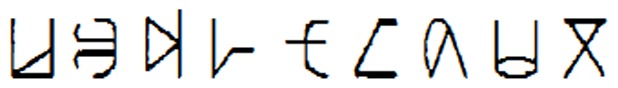
The nine Gibson figures.

In the present study, we used nine Gibson figures (see Figure 1) [23]. Subjects who had no prior exposure to these figures were asked to learn to associate them either with dot numerosity (the magnitude-learning [ML] group) or with spatial order (the order-learning [OL] group). Detailed procedures for the training are described in the method section and Appendix S1. Event-related potentials (ERPs) were recorded while subjects performed magnitude comparison tests after each session of training.

Although the above design would allow us to examine similarities and differences in the learning of magnitude and spatial order, it did not rule out the possibility that magnitude learning might have involved certain order learning (e.g., serial order in magnitude). To examine whether information about order (any type of order) is a necessary component of the learning of magnitude, we used the N400 paradigm based on the pre- and post-training ERP. N400 has been extensively used as an index of semantic priming in ERP research on language because it is sensitive to semantic violations of sentences or unrepeated word lists [24]. In addition, N400 or an N400-like component's [25], [26] amplitude is also sensitive to incorrect answers to arithmetic equations [27], [28], especially when the incorrect answers are far away from the correct ones [29], [30]. Of most relevance to the present study, N400 is sensitive to sequential order of numbers [31], with a larger N400 for out-of-order lists than for sequentially ordered lists. In our study, we used the N400/N400-like component to investigate whether magnitude- (ML) and/or spatial order-learning (OL) resulted in the learning of sequential order of the artificial symbols. We expected that the OL group would show an N400/N400-like effect (i.e., the ordered lists would elicit a smaller N400/N400-like component than would the out-of-order lists) because spatial order has an inherent sequential order. For the ML group, the two hypotheses of mental representations of numbers would make different predictions. If the mental number line hypothesis is correct, order information would be acquired as part of magnitude learning and the ML group would show the N400/N400-like effect; however, if the numerosity code hypothesis is correct, magnitude learning would not involve order information and the ML group would not show an N400/N400-like effect.

## Materials and Methods

### Ethics statement

This study was approved by the Institutional Review Board of BNU Imaging Center for Brain Research. All participants were volunteers and signed informed consent.

### Subjects

Fifty-nine healthy and right-handed college students participated in the experiment. They were divided into two groups, matched in gender, age, and school major. One group was assigned to magnitude learning (ML), and the other to spatial-order learning (OL). Data from four subjects were discarded because of excessive eye-blinking during ERP recording. Another subject was excluded because he showed only chance level performance during both learning and test phrases. The final usable sample included 27 subjects in the ML group (13 males, mean age = 21 yrs, with a range of 17–26 yrs) and 27 subjects in the OL group (14 males, mean age = 20.4 yrs., with a range of 17–24 yrs).

### Materials

**Figure 2:**
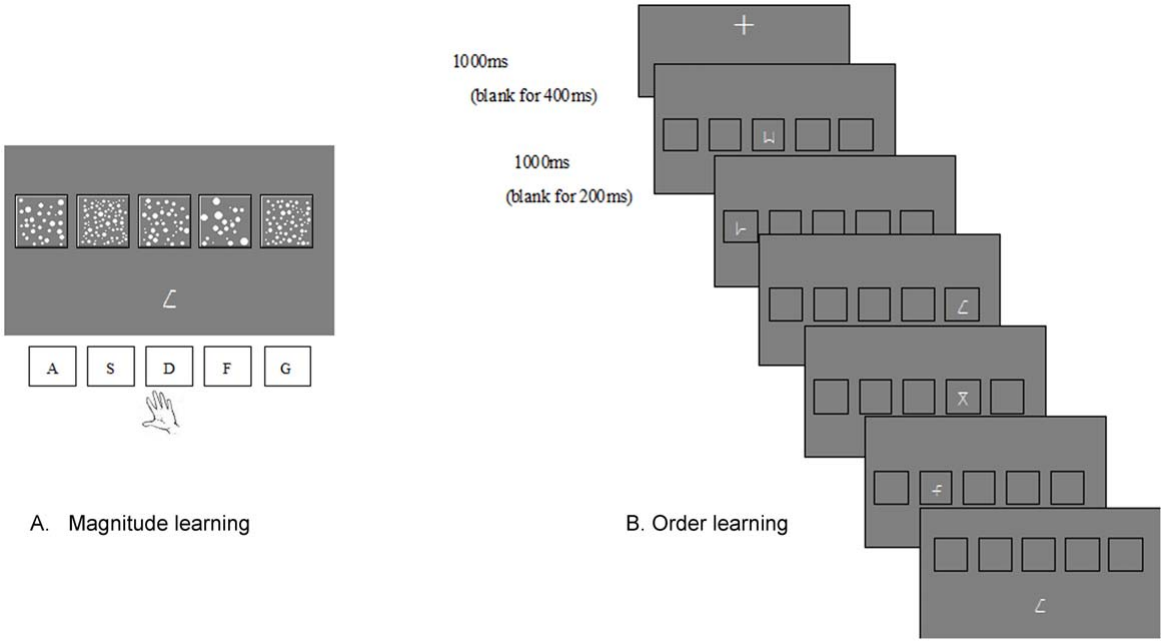
Experimental design and sample stimuli for the reviewing unit. (a) Magnitude Learning (b) Order Learning.

Nine Gibson figures used in Tzelgov's experiment [23] were used as the artificial numbers/objects. The figures were associated with either numerosity or spatial order depending on the learning groups. The ML subjects would learn to associate the nine Gibson figures with the dot patterns of 10, 20, 30, 40, 50, 60, 70, 80 and 90 dots respectively. The dots were fitted into a frame of the size 2.8 cm×2.75 cm. The total surface area of all dots combined was fixed by varying the size of dots both within (randomly distributed) and across patterns (see Figure 2 for samples). The OL subjects were asked to learn the relative order of the nine symbols by seeing a sub-set of them presented in relative order on five positions from left to right. The five positions were marked by black-framed squares of 2.8 cm×2.75 cm in size.

### Experimental Design and Procedure

**Figure 3:**
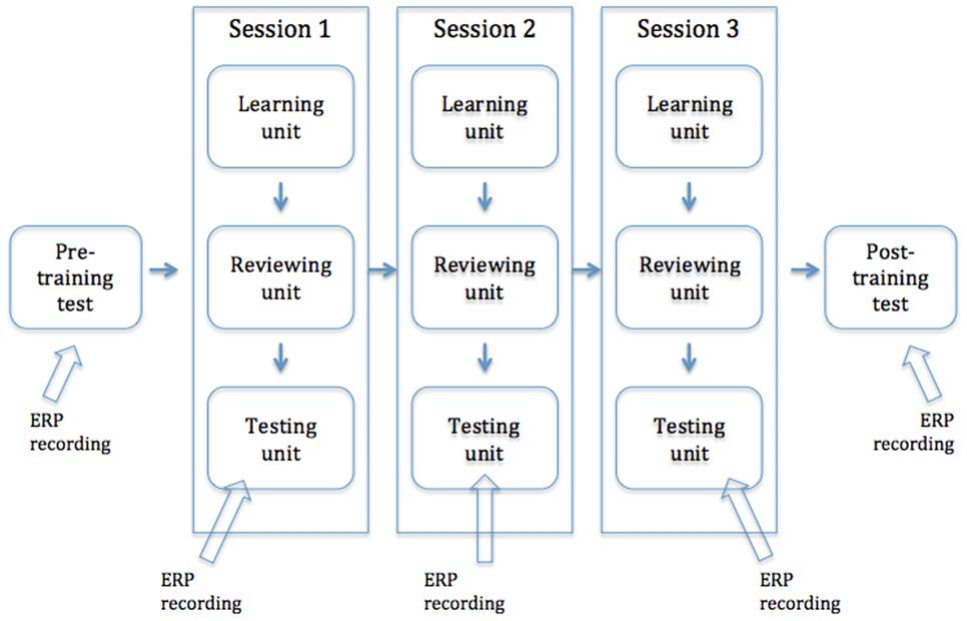
Schematic representation of the experimental procedure.

**Figure 4:**
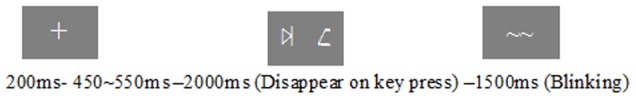
Task design of the test unit.

Both ML and OL conditions included a pre-training test, three training sessions and a post-training test (see Figure 3). Each training session had three units: learning, reviewing, and testing. In the learning unit, subjects in the ML group were asked to remember the symbols and their associated approximate numerosities and subjects in the OL group learned relative spatial order of the nine symbols. After the learning unit, subjects then reviewed the materials in the reviewing unit (Figure 2). In the testing unit, subjects were presented a pair of symbols (Figure 4) and were asked to judge which was larger in magnitude in the ML group and which was to the right of the other symbol in the OL group. The stimulus pairs included both far- and close-distance pairs (see Appendix S1 for details). Subjects responded by pressing a key as fast and accurately as possible. There was a break after every 59 stimuli. There was one practice trial before each test to ensure that subjects understood the procedure. ERP data from the testing units were used to examine P2p and N2 components (i.e., the distance effect). Details for training and testing are described in Appendix S1.

**Figure 5:**
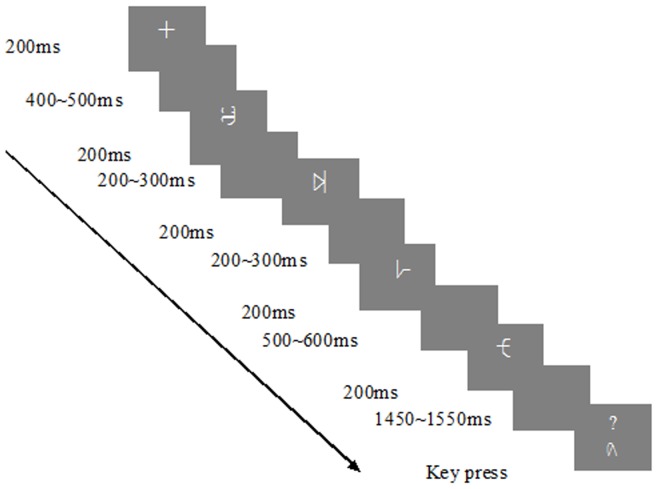
Task design of the pre- and post-training test.

To examine the N400/N400-like component to see whether subjects obtained the order information, a 2 groups (ML and OL)×2 sessions (pre- and post-training tests) design was used (Figure 5). For this test, subjects were presented five symbols sequentially under two conditions. For the “ordered” condition, the first four symbols were presented in the order as learned during the training (i.e., from small to large for the ML group or from left to right for the OL group). For the “unordered” condition, the first four symbols were presented in a random order. In order to control for potential N400/N400-like differences that might be induced by different numerical distances between the third and fourth stimulus, we fixed the distance between the two stimuli to “1” (i.e., the neighboring items in numerosity or spatial order). Because we modified the N400 paradigm, we will term our component as the N400-like component. The fifth symbol was used to keep subjects' attention and related ERP were not analyzed. For more details, see Appendix S1.

### ERP Recording and Analysis

EEG was collected using a Quick-cap with 64 silver chloride electrodes of the Neuroscan system. All electrodes except for EOG were physically referenced to the left mastoid and were then re-referenced off-line with “average mastoids reference derivation” to have a linked-mastoids reference [32]. Signals were amplified using a band pass of 0.05–100 HZ followed by a 50-Hz notch filter and were digitized using a 16-bit A/D converter at 500 Hz sample rate. Electrode impedance was kept below 5 kΩ. EEG data were processed off-line using Scan. Following the procedure recommended by Luck [33], (p. 157), we conducted artificial rejection of trials in two steps. First, trials contaminated by eye blinks or other artifacts were rejected according to the rejection criteria of ±70 µv for all channels. Second, when too many trials (over 20%) would have to be deleted just because of contamination of eye movement for a particular subject, we would conduct ocular artifact reduction specifically for that subject and then conduct artificial rejection with the criteria of +/−45 uv except for VEOG.

Subjects sat in a quiet and appropriately illuminated room. There was 80 cm between the subject and the screen. Each stimulus included a pair of symbols, whose centers were 3.5 cm apart. The stimulus was 2 cm high and 5 cm wide, with a horizontal visual angle of 3.6° and a vertical angle of 1.4°. Subjects were asked to blink only after each epoch period. Each test lasted for 7 to 8 minutes, with a short break after every 2 or 3 minutes.

ERPs were time locked to the onset of the stimulus, with a 200 ms pre-stimulus baseline. When analyzing the ERP data from the three testing units during training, we selected two ERP components based on previous studies of the numerical distance effect, P2p [21], [22], [34] and N2 [21]. The mean amplitude of P2p was computed in the window of 250–310 ms, on the posterior electrode groups (parietal: P3/P4, P5/P6; occipital: PO5/PO6, O1/O2). The mean amplitude of N2 was computed in the time window of 310–380 ms, on representative electrodes for the frontal (F3/F4, F5/F6), central (C3/C4, C5/C6), parietal (P3/P4, P5/P6) and occipital (PO5/PO6, O1/O2) groups. The scalp electrodes were grouped in terms of their locations in the anterior-to-posterior direction (frontal, central, parietal and occipital) and hemisphere (left and right). Voltages averaged over those electrode groups and time windows were then entered into repeated measures ANOVA with the Greenhouse-Geisser correction for nonsphericity [35]. Distance (close, far), session (test 1, 2, 3), electrode position (parietal and occipital for P2p; frontal, central, parietal and occipital for N2) and hemisphere (left, right) were the within-subject factors and group (ML, OL) was the between-subject factor. Bonferroni correction was used.

The N400-like effect was observed only in the post-training test for the OL group. It was a negative-going component that was prevalent over the frontal-central areas. It started around 300 ms after the presentation of the fourth symbol, peaking around 350–400 ms, and dropping down around 550 ms. To compare this component across the conditions and sessions, we calculated average amplitude during the time window of 300–550 ms at the frontal-central (FC3/FC4, FC5/FC6) and central electrodes (C3/C4, C5/C6). They were analyzed with 2 (session: pre and post)×2 (order-priming: ordered, unordered)×2 (electrode position: frontal-central, central)×2 (hemisphere) repeated measures ANOVA. These analyses were done separately for the two learning groups.

## Results

### Behavioral Results

**Figure 6:**
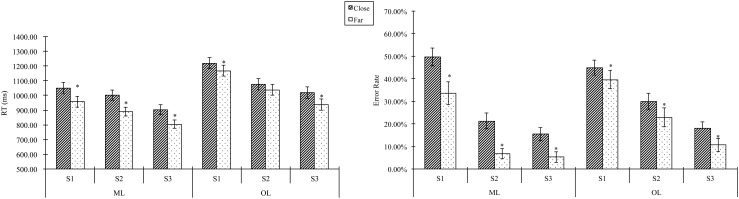
Behavioral distance effect across three tests during training. ML: Magnitude learning; OL: Order learning; S1, 2, 3: Session 1, 2, 3.

Both error rate and reaction time data were subjected to 2 (distance)×3 (session)×2 (learning group) repeated measures ANOVAs, with the group as the between-subjects factor. As shown in Figure 6, the RTs of all three tests in the ML group (1000 ms, 944 ms and 852 ms respectively) were faster than those in the OL group (1180 ms, 1063 ms, 978 ms) [F(1, 50) = 9.586, P = 0.003]. The two groups did not differ significantly in error rates (ER). The learning effect was shown by decreasing RT [F (2, 49) = 43.856, *P*<0.001, ε = .811] and ER [F (2, 49) = 90.516, *P*<0.001, *ε* = .870] across the three training sessions. Furthermore, there was a significant interaction between session and group for ER [F (2, 49) = 3.678, *P* = 0.029]. The ER decreased dramatically in the second session for the ML group, but more gradually for the OL group. Specifically, the ER across the three training sessions were 41.5%, 13.6% and 10.1% for the ML group, and 42.1%, 26.3%, 14.2% for the OL group.

The distance effect was significant for both RT [F (1, 50) = 94.590, P<0.001, ε = .870] and ER [F (1, 50) = 69.863, P<.001]. There were also significant interactions between the distance effect and the learning group [RT: F (1, 50) = 7.298, P = .009; ER: F (1, 50) = 7.908, P = .007], with greater differences between the two learning groups under the far-distance condition than under the close-distance condition.

### ERP Results from the Three Tests Administered During the Three Training Sessions

**Figure 7:**
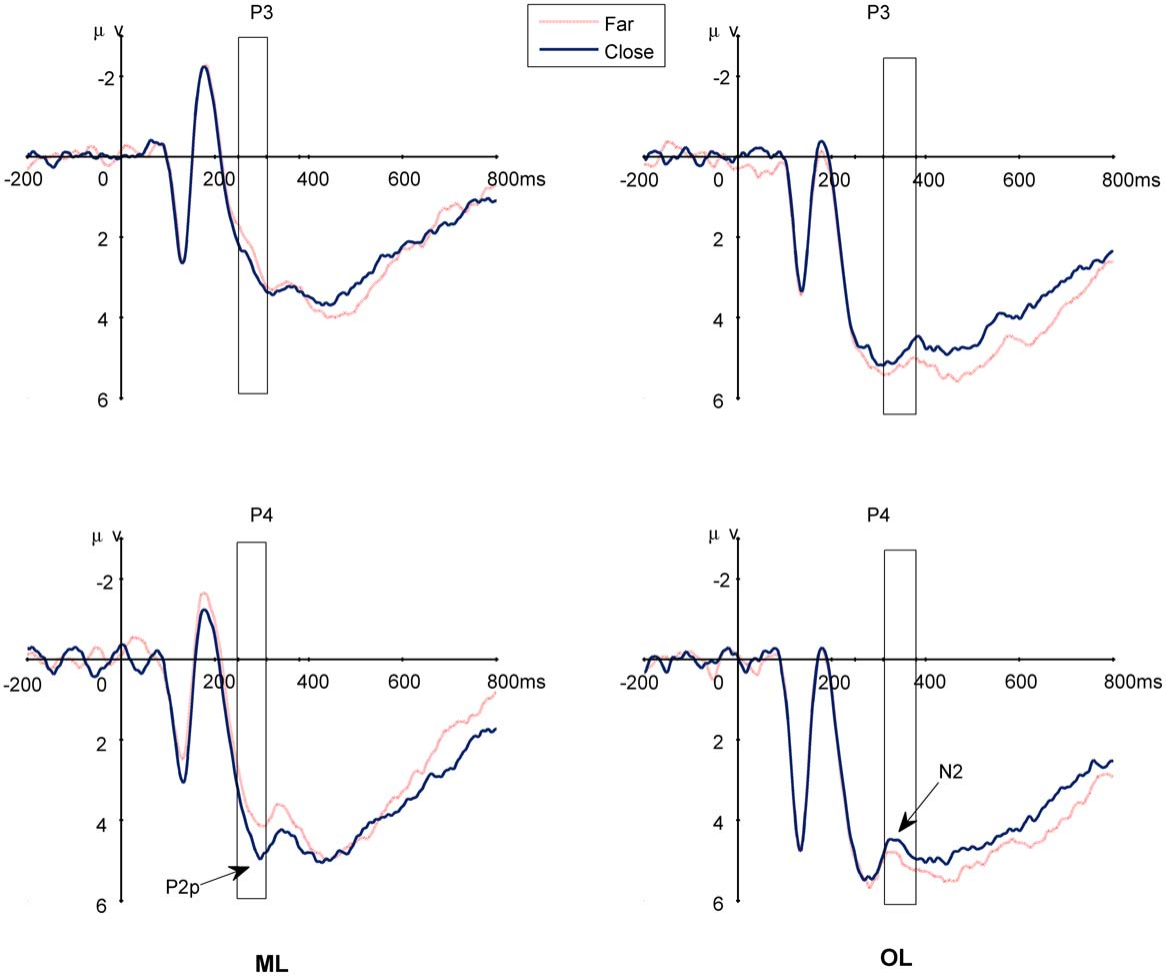
The grand average ERPs elicited by far- and close-distance pairs in Session 3. The rectangles indicate the time windows analyzed. The distance effect in the ML group appeared earlier on P2p, but later on N2 for the OL group. The pattern of the distance effect was opposite between the two groups. ML: magnitude learning; OL: order learning.

**Figure 8:**
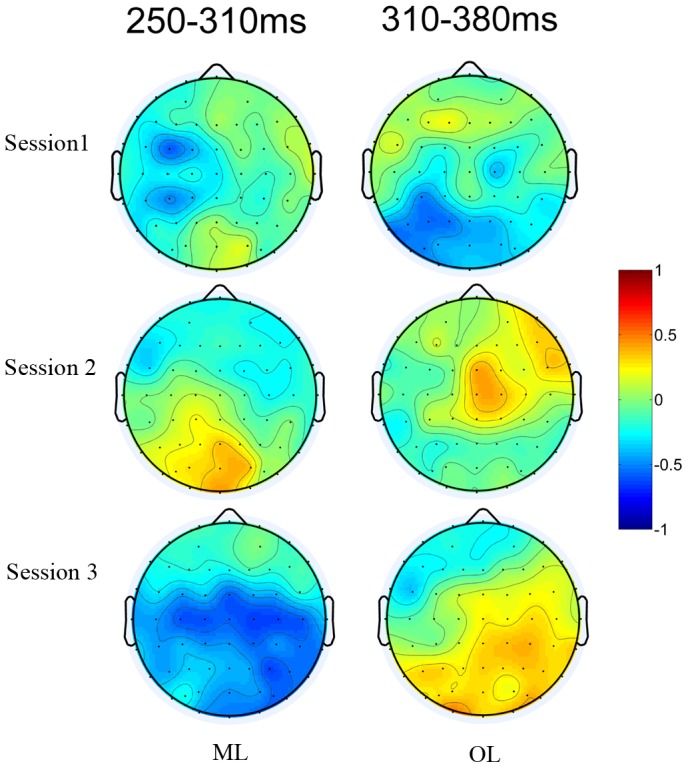
Topography of changes in the distance effect across the three tests administered during the three training sessions. The distance effect (far distance – close distance) over the time windows of 250–310 ms and 310–380 ms for the ML and OL groups.

Guided by previous research as reviewed in the introduction section, we focused our analysis on the P2p and N2 components. Figure 7 shows the grand average ERP waveforms of Session 3. Figure 8 shows the topography of the distance effect across the three training sessions.

#### The P2p component

Mean amplitudes of P2p were analyzed with a repeated measures ANOVA with session, distance, and electrode group as the within-subjects factors and learning group as the between-subjects factor. The main effect of session was significant (F (2, 104) = 5.874, P = 0.004). Post hoc analysis with Bonferroni correction indicated that the mean amplitude was significantly higher for Session 1 than for the other two sessions. The main effect of distance (F (1, 52) = 4.922, P = 0.031) and electrode group (F (1, 52) = 15.959, P<0.001) were also significant. There were also a two-way interaction between session and electrode group (F (2, 104) = 3.130, P = 0.048), a three-way interaction among distance, session and group (F = 3.572, P = 0.032), and a four-way interaction involving all four variables (F (2, 104) = 6.101, P = 0.003). We next describe each of these significant interactions.

Post hoc analysis of the two-way interaction showed that the mean amplitude was significantly higher for Session 1 than for Sessions 2 (P = 0.002) and 3 (P = 0.019) at the parietal sites, but only significantly higher than Session 2 (P = 0.032) at the occipital sites.

Of most relevance to our research question, post hoc analysis of the three-way interaction showed that the distance effect was significant for Session 1 for the OL group (P = 0.038), but significant for Session 3 for the ML group (P = 0.023). In both cases, the close-distance trials elicited a larger positivity than the far-distance trials.

**Figure 9:**
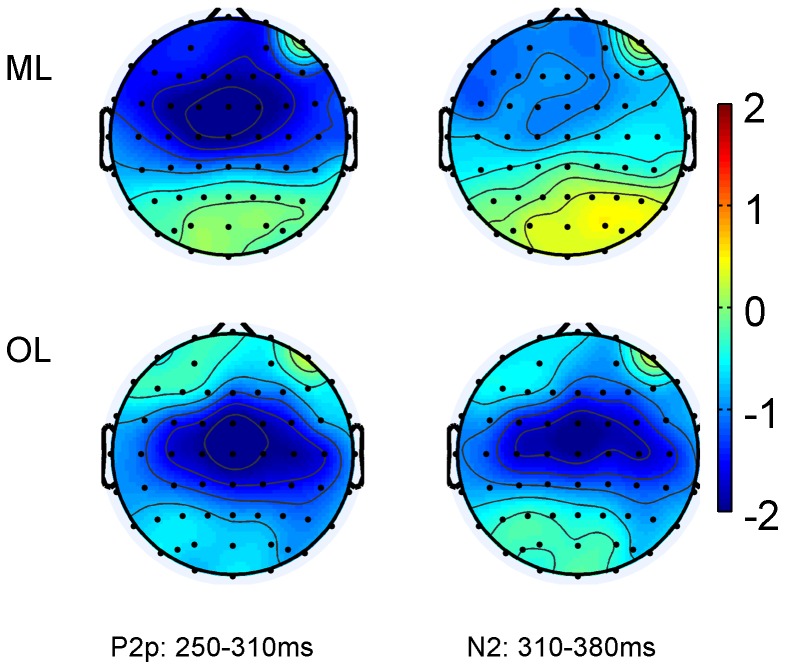
Topography of the general learning effect: Differences between Session 3 and Session 1 in P2p (250–310 ms) and N2 (310–380 ms).

In terms of the four-way interaction, post hoc analysis showed that the effect of session (or the learning effect) was bilaterally distributed for the OL group (left: P = 0.041; right: P = 0.009) but right lateralized for the ML group (P = 0.036) at the parietal sites. At the occipital sites, the learning effect was significant on the left hemisphere for the OL group (P = 0.028), and marginally significant on the right hemisphere for both groups (ML: P = 0.059; OL: P = 0.053) (see Figure 9).

To examine whether the faster reaction time of the ML group than the OL group might have contributed to the earlier appearance of the distance effect in the ML group (Session 3), we reran the analyses by excluding the top 10% fastest subjects of the ML group and the bottom 10% (i.e., slowest) of the OL group to match the behavioral performance of the two learning conditions. For the subsamples (24 subjects for each group), the mean RTs in test 3 were 881.05 ms for the ML group and 937.93 ms for the OL group, which did not differ significantly (P = 0.179). Results showed that the distance effect still appeared earlier in the ML group than in the OL group for P2p at the parietal and occipital sites (distance×group: F = 4.771, P = 0.034).

#### The N2 component

The main effects of session [F (2,104) = 8.799, P = 0.001, ε = 0.794] and electrode [F (3,156) = 60.446, P<0.001, ε = 0.449] were significant. Post hoc analysis with Bonferroni correction indicated that the mean amplitude was significantly lower for Session 1 than for the other two sessions. The mean amplitude of N2 was highest at the frontal sites, followed by the central, occipital and parietal sites in that order.

Two-way interactions between electrode and other factors were significant: group [F (3,156) = 6.054, P = 0.01, ε = 0.449], session [F (6,312) = 4.977, P = 0.006, ε = 0.391] and hemisphere [F (3,156) = 7.432, P = 0.001, ε = 0.751]. Post hoc analysis of the two-way interactions between electrode and group showed that the mean amplitude of N2 was significantly larger at the occipital sites (P = 0.026) and marginally larger at the parietal sites (P = 0.07) for the ML group than for the OL group. In terms of the interaction between electrode and hemisphere, the mean amplitude of N2 was higher in the right hemisphere than in the left hemisphere at the frontal sites (P = 0.015), but did not differ at other sites. In terms of the interaction between electrode and session, the mean amplitude became higher across the three sessions at the frontal and central sites, whereas the highest amplitude occurred during the second session at the parietal and occipital sites.

There were also a significant three-way interaction among session, hemisphere and group [F (3,156) = 5.835, P = 0.006, ε = 0.862] and two significant four-way interactions of session×distance×group×electrode [F (6,312) = 3.572, P = 0.032, ε = 0.447] and session×distances×electrode×hemisphere [F (6,312) = 2.482, P = 0.042, ε = 0.696]. The significant three-way interaction was due to the face that the mean amplitude of the three sessions changed bilaterally for the OL group (P = 0.004 for the left hemisphere, and P<0.001 for the right hemisphere), but only changed in the right hemisphere for the ML group (P = 0.018)(Figure 9.)

The most important results were the four-way interactions. Post hoc analysis on the interaction among session, distance, group and electrode indicated that in the first session, the only significant distance effect was in the OL group at the parietal (P = 0.039) and occipital sites (P = 0.027), with a larger N2 for the far-distance trials. In the third session, the distance effect was significant for the OL group (P = 0.039), but marginally significant for the ML group (P = 0.067) at the occipital sites. The close distance trials elicited larger N2 in the OL group, which was reversed as compared to the previous sessions and that of the ML group. Post hoc analysis of the other significant four-way interaction among session, distance, electrode and hemisphere showed that the amplitude of N2 was right lateralized at the frontal sites for the close-distance trials during all three sessions, but left lateralized at the parietal sites for the close-distance trials in Session 3.

### The Order-Priming Effect: the N400-like component from Pre- and Post-Training Tests

**Figure 10:**
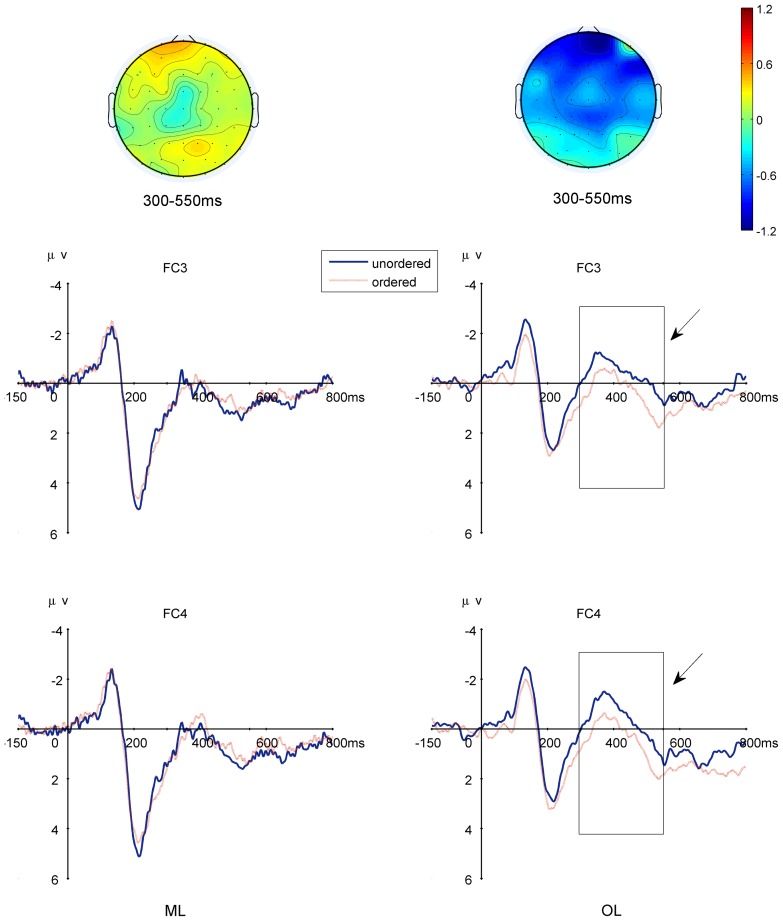
N400-like priming effect in the post-training test over the left and right frontal-central electrodes. Upper: Topography of the N400-like priming effect for the two groups: Lower left: FC3; Lower right: FC4. Note that the priming effect was only significant for the post-training test of the OL group.

Repeated measures ANOVA showed that the interaction of session×order-priming was significant only for the OL group [F(1,26) = 4.529, P = 0.048]. Post hoc analysis showed a significant order-priming effect for the post-training test (P = 0.027), in which the ordered condition elicited a smaller N400-like component (Figure 10) than did the unordered condition. There was no order-priming effect for the ML group on both of the pre- and post-training tests at any of the brain areas: Neither the main effect of order-priming (P>0.8) nor the interaction of session×order-priming (P>0.8) was significant.

## Discussion

Using an artificial symbol training paradigm and the ERP technique, the current study investigated the underlying neural mechanisms of magnitude and order learning. Results showed different ERP time courses for the learning and representations (i.e., the distance effect) of magnitude and order. Furthermore, by examining the N400-like component, our study also provided direct evidence that magnitude representation does not have to entail order information. In the following paragraphs, we discuss our findings in the context of the existing literature and theoretical models of mental representations of numbers.

### Temporal and topographical differences between the representations of magnitude and spatial order

First, we found that the ERP distance effect appeared earlier in the ML group than it did in the OL group. This result is consistent with a previous study that used natural numbers [22]. Turconi et al. [22] also found that the distance effect appeared around 170–210 ms for quantity comparison of numbers but 210–240 ms for order comparison of numbers. One explanation of the earlier distance effect of magnitude learning is that judgment of a given numerosity involves only the retrieval of its representation (or numerosity code), whereas order judgment involves the retrieval of two codes and their relative order.

Another important difference in ERP between the learning of magnitude and order is the reversed pattern of the ERP distance effect. Close-distance stimuli elicited a larger positivity (P2p) in the ML group, but a larger negativity (N2) in the OL group. Our finding of the distance effect in the early part of P2p for magnitude learning is consistent with previous studies that such as Turconi et al. [22], Szűcs & Csépe [21], and Dehaene [34].Although the time window was somewhat later perhaps due to the slower processing of the newly learned materials. Moreover, the reversed distance effect in the OL group is similar to the distance effect found for letters on N2p by Szűcs and Csépe [21]. One explanation of this reversed distance effect is that the order comparison task involves a search pattern strategy. It should be noted, however, Turconi et al. [22] did not find such a reversed distance effect when they used a numerical order task, perhaps because that task involved both order and magnitude.

The present study also revealed differences in hemispheric asymmetry of magnitude and order learning. The training effect (indexed by a reduced P2p and an increased N2) was right lateralized for magnitude learning, but bilateral for order learning. Turconi et al. [22] also revealed a bilateral distance effect for order processing, but they found left lateralization for magnitude processing. Similarly, Lyons and Ansari [36] also found left lateralization in their study of artificial digit learning. It appears that the role of the right hemisphere in magnitude learning is inconsistent. Other researchers have argued that the right parietal region is especially sensitive to Arabic digits and dot arrays as compared to other formats of numbers such as number words [37], [38]. Prado et al. [39] also found activation at the right IPS for magnitude comparison in addition to the commonly reported activation in the left IPS elicited by both magnitude and spatial order transitive tasks. Given these inconsistent results regarding the role of the right hemisphere in magnitude learning and processing, future research should use a broader array of magnitude-related tasks. Another possible reason for our finding of generally rightward bias in laterality may involve the way Chinese language is processed. Some studies have shown that the right hemisphere plays an important role in Chinese language processing [40], [41], [42].

### Implications of the order-priming effect as revealed by the N400-like component

In the order-priming experiment, we found that the ordered lists elicited a smaller N400-like component than did the unordered lists after the OL group learnt the order of the stimuli. In contrast, the ML group did not show the order-priming effect, suggesting this group did not derive order information from magnitude learning. This order-priming effect is similar to previous studies of (dis)order effects in numerical sequences [31], addition [29], [43] or multiplication equations [27], [28], and semantic violations in sentences [44], [45]. It should be noted that different studies have found different locations of the N400 or N400-like component perhaps due to the differences in materials and tasks. Many studies found the effect in the central-parietal areas, but several studies including our own have shown an anterior distribution of the effect. For example, Galfano et al. also found a greater N400-like effect in the frontal area during a number-matching task [25], [26]. Similarly, Zhou et al. [30] found a frontal-central distribution of the N400-like component with a different number matching task. Interestingly, the topography showed to a frontal–central shift when the numbers were presented sequentially in an addition task [29], [43] as compared to being presented simultaneously as equations [46]. These anterior effects appears similar to the anterior N400 elicited by semantic priming tasks involving line drawing and object matching tasks [47], [48]. Therefore, there appears accumulating evidence confirm the claim of N400 as the index of general semantic intergration regardless of the stimulus material [31], albeit with slightly different topographic distributions. Future research is needed to directly examine the effects of task design (e.g., task-relatedness and the strength of semantic context) on the distribution of the N400 effect.

We interpret our results from the N400-like effect as direct evidence that order was not an inherent attribute of magnitude. The ML group did not appear to have acquired order information after three sessions of learning. These subjects were able to compare the magnitude of numbers without processing their order information. In other words, magnitude information does not need contain spatial order. This of course does not imply that the ML group would never learn the order information. Given time, one assumes that these subjects would begin to incorporate other features such as order into magnitude learning. Our claim is that with proper design we were able to separate the learning of magnitude from that of order and, through such a separation, we found that subjects could represent magnitude without using order information in a short-term experimental session. Future research should examine whether in the long run the order information can also be separated from the magnitude representation.

### Implications for the magnitude representation hypotheses

Taken together our results, there is strong evidence for the numerosity code hypothesis of number representations. According to this hypothesis, magnitude is represented straightforwardly as the number of units activated, and the bigger numbers include smaller numbers as subsets [9]. The distance effect was interpreted by the difference in activated nodes between the closer number pairs and the farther number pairs. During a comparison task, subjects would compare two symbols on the basis of retrieved representations. Such a task is easier than order judgment. Thus the learning of magnitude can be fast. Because magnitude learning is relatively easy and it does not have to involve learning order information, human infants and animals are able to distinguish different magnitudes without understanding numerical order, although they show the basic ability to process both magnitude and general order information [49]. We should hasten to add that, although our evidence is consistent with the numerosity code hypothesis, our conclusions are restricted to artificial symbol learning and are likely to reflect initial learning of numbers by infants or young children. The contrasting hypothesis of number representations—the mental number line hypothesis—is more likely to reflect the combined attributes of both cardinality and ordinality of natural numbers. The orientation of the number line might be acquired during later learning of number sequence, which led to context-dependent SNARC effect [12], [50]. Future research should consider a longer-term training design and integrate the training of both ordinality and cardinality (either simultaneously or sequentially). Finally, the present study only focused on the different views about the role of order information in magnitude representation between the two hypotheses. Future research should examine other aspects of differences (e.g. the pattern of activated nodes, dimensions of the representation, etc.) between the two hypotheses.

### Conclusion

By using an artificial symbol learning paradigm and the ERP technique, the present study revealed differences in neural mechanisms underlying the learning and representation of magnitude and spatial order. The distance effect appeared earlier in the magnitude-learning group than the order-learning group. The close-distance trials were more positive than the far-distance trials in the magnitude-learning group, whereas the pattern was reversed in the order-learning group. Finally, order-priming data showed that order information was not inherent to magnitude learning. These results support the numerosity code hypothesis.

## Supporting Information

Appendix S1 Details of the three units of training: learning, reviewing, and testing.
(DOC)
