# Supplementary material for: Is Order the Defining Feature of Magnitude Representation? An ERP Study on Learning Numerical Magnitude and Spatial Order of Artificial Symbols
Source: PLoS One. 2012 Nov 19;7(11):e49565. doi: 10.1371/journal.pone.0049565 (PMC3501518; doi:10.1371/journal.pone.0049565)
Supplement: Appendix S1 — Details of the three units of training: learning, reviewing, and testing. (DOC) [file pone.0049565.s001.doc]

Appendix:

Details of the three units of training: learning, reviewing, and testing.

The learning unit: In the learning unit for the ML group, a symbol was displayed in the center of the screen, above which was a dot pattern. Subjects were asked to remember the symbol and its associated approximate numerosity. Each symbol-dot-pattern pair was presented for a total of 15 times (each time for 1000ms followed by a 100ms blank). The 15 presentations were organized into five clusters of three presentations each.

For the OL group, five symbols would appear one by one in a random temporal order in the five framed, horizontally arranged, positions on the screen. The reason to use five positions for the subjects to learn nine symbols is that this would force subjects to learn relative spatial order of the nine symbols, not just spatial positions (which would have been the case if nine positions/frames had been used). For each trial, the five symbols were pseudo-randomly selected from the nine symbols. The use of pseudo-random selection was to ensure that each symbol was presented 15 times in five clusters, just as in the ML group. Although different sets of five symbols were selected for each trial, the nine symbols’ relative order was the same so subjects could learn the spatial order of the symbols. Of course, subjects had to abstract the sequence of the nine symbols’ spatial positions based on their relative spatial relations from each presentation of five symbols.

The reviewing unit: In the reviewing unit for the ML group, five dot patterns (randomly selected from the nine patterns subjects learned) were presented simultaneously in top half of the screen and a symbol corresponding to one of the patterns was presented below them. Subjects were asked to choose the dot pattern that matched the symbol by pressing one of five corresponding keys (see Figure 3a). For the OL group, subjects would first see five symbols (randomly selected from the nine symbols they learned) one by one on the screen. They appeared in their relative spatial order in the boxes (i.e., in the order they were trained). The temporal order of the presentation of the five symbols was random (see Figure 3b for an example). Finally, subjects were presented with five black-framed squares on the top half of the screen and one of the five symbols on the bottom half. They were asked to press a key to indicate to which square the symbol belonged (Figure 3b). In the reviewing unit, each combination of symbol-dots and symbol-position was repeated five times. Subjects were told to focus on accuracy, not speed. Feedback was provided after each trial.

The testing unit: To examine the distance effect, the pairs of symbols were divided into two groups with different numerical/spatial distances. A difference of 10-30 dots in the ML group and 1-3 spatial positions in the OL group was considered as “close” distance, and a difference of 40-80 dots and 4-8 positions as “far” distance. In order to control the number of test trials and the use of each symbol in both distance groups, we chose four pairs from each distance group as the test material. For the ML task, the close-distance pairs were 20-30, 30-40, 60-80, and 70-80; and the far-distance pairs were 20-60, 30-70, 30-80, and 40-80. Accordingly, the close-distance pairs in the OL group were 2-3, 3-4, 6-8, and 7-8, and the far-distance pairs were 2-6, 3-7, 3-8, and 4-8. The display positions of the pairs were counterbalanced (e.g. 2-6 and 6-2). Each of the eight pairs (four pairs x two display positions) for each distance was repeated eight times, yielding a total of 64 stimuli in each distance type. In order to avoid the potential “endpoint effect” (i.e., subjects might easily recognize the first and the last symbol and made judgment of order without actually comparing the pairs), eight filler pairs were included. They were created by pairing symbols for 10 and 90 for the ML group and symbols for 1 and 9 for the OL group with another symbol. The eight filler pairs were repeated six times to yield 48 trials. Altogether, there were 176 pairs for every test (64 close-distance and 64 far-distance pairs).

Details of the pre- and post-training tests

These tests were used to examine the order effect as revealed by the N400 component. In both pre- and post-training tests, subjects were presented five symbols sequentially after a 200ms fixation “+” for each trial. The first four symbols (presented for 200ms each) either followed the order based on the training (from small to large for the ML group or from left to right for the OL group), which was the “ordered” condition, or were randomized, which was the “unordered” condition. (It should be noted that the order information was based on the training, thus irrelevant for the pre-training test. But to allow for comparisons, we used the same design for both pre- and post-training tests.) To reduce potential N400 differences that might be caused by the different distances between the third and the fourth symbols for the two conditions, these two symbols were exactly the same across the two conditions. For example the unordered trials would be 2-6-3-4 and 1-5-7-8, whereas the corresponding trials for the ordered condition were 1-2-3-4 and 5-6-7-8, where the numbers denote the sequential positions of the symbols during training. To maintain subjects’ attention (especially in the ordered condition), a fifth symbol was presented for 1500ms after the fourth symbol. The fifth symbol was randomly selected and appeared underneath a “?” mark. Subjects were asked whether this symbol was included in the previous four symbols. This verification task was used to encourage subjects to concentrate on the stimuli when viewing the first four symbols. Its ERP data were not analyzed. The delay of 1500ms was to allow us to observe the N400 effect from the list of the first four symbols. The fifth symbol disappeared after the subjects responded by pressing a key or two seconds had lapsed. Both the ordered and unordered conditions had 48 trials (12 sequences repeated four times each). There was a break after every 32 trials. ERPs were recorded online. ISI was randomly jittered. Please refer to Figure 5 for more details.
